# Low magnesium in conjunction with high homocysteine increases DNA damage in healthy middle aged Australians

**DOI:** 10.1007/s00394-024-03449-0

**Published:** 2024-06-12

**Authors:** Varinderpal S. Dhillon, Permal Deo, Michael Fenech

**Affiliations:** 1 Health and Biomedical Innovation, UniSA Clinical and Health Sciences, University of South Australia, Adelaide, 5000 Australia; 2 Genome Health Foundation, North Brighton, 5048 Australia

**Keywords:** Magnesium, Homocysteine, DNA damage, Cytokinesis-block Micronucleus Cytome, Micronuclei (MN), Nucleoplasmic bridges (NPBs), Nuclear buds (NBuds)

## Abstract

**Purpose:**

Magnesium is one of the most common elements in the human body and plays an important role as a cofactor of enzymes required for DNA replication and repair and many other biochemical mechanisms including sensing and regulating one-carbon metabolism deficiencies. Low intake of magnesium can increase the risk of many diseases, in particular, chronic degenerative disorders. However, its role in prevention of DNA damage has not been studied fully in humans so far. Therefore, we tested the hypothesis that magnesium deficiency either on its own or in conjunction with high homocysteine (Hcy) induces DNA damage in vivo in humans.

**Methods:**

The present study was carried out in 172 healthy middle aged subjects from South Australia. Blood levels of magnesium, Hcy, folate and vitamin B_12_ were measured. Cytokinesis-Block Micronucleus cytome assay was performed to measure three DNA damage biomarkers: micronuclei (MN), nucleoplasmic bridges (NPBs) and nuclear buds (NBuds) in peripheral blood lymphocytes.

**Results:**

Data showed that magnesium and Hcy are significantly inversely correlated with each other (*r* = − 0.299, *p* < 0.0001). Furthermore, magnesium is positively correlated both with folate (*p* = 0.002) and vitamin B_12_ (*p* = 0.007). Magnesium is also significantly inversely correlated with MN (*p* < 0.0001) and NPB (*p* < 0.0001). Individuals with low magnesium and high Hcy exhibited significantly higher frequency of MN and NPBs compared to those with high magnesium and low Hcy (*p* < 0.0001). Furthermore, there was an interactive effect between these two factors as well in inducing MN (*p* = 0.01) and NPB (*p* = 0.048).

**Conclusions:**

The results obtained in the present study indicate for the first time that low in vivo levels of magnesium either on its own or in the presence of high Hcy increases DNA damage as evident by higher frequencies of MN and NPBs.

## Introduction

A diet rich in essential micronutrients is key to better health and wellbeing and lowers the risk of developmental defects and chronic degenerative diseases [1, 2]. Improved infant, child and maternal health, stronger immune system, lower risk of non-communicable diseases, and longevity can be attributed to better nutrition. Damage to nucleic acids such as DNA can happen at any stage of life (starting from conception to old age) and has detrimental effects on development and organ function due to acquired mutations [3–5]. DNA damage events such as cells with multicentric chromosomes, micronuclei, or extremely short telomeres can lead to genomic instability [6].

Magnesium is the 4th most abundant mineral present in human body and is involved as a cofactor in major metabolic and biochemical pathways within the cell [7–9]. It is associated with various functions within the body such as strengthening and development of bones, nerve function, regulating blood sugar and blood pressure [10], protein metabolism, nucleic acid stability (DNA and RNA), and cell proliferation [9]. More than 600 enzymes require magnesium as cofactor and almost 200 require it as an activator [11, 12]. In addition to these, magnesium also plays an important role in DNA repair mechanism pathways as many enzymes such as DNA polymerase beta, DNA ligases, and DNA endonucleases requires magnesium for their proper functioning [13]. Adequate level of magnesium is required for efficient DNA replication and DNA repair both of which are essential for maintaining genomic stability [13, 14]. It has been recently shown that low levels of magnesium are associated with shorter telomere length and less sleep [15].

Homocysteine (Hcy) is metabolized from methionine obtained from diet and its elevated blood level is associated with increased risk for neurodegenerative diseases such as dementia, Alzheimer’s and Parkinson’s disease, and neural tube defects [16–20]. Accumulation of DNA damage can elicit excessive apoptosis or cell death of neurons thereby leading to neurological diseases [21–24]. Homocysteine is elevated by deficiency of folate and vitamin B_12_ because they are required to convert homocysteine back to methionine [25] and high homocysteine may increase DNA damage by impairing FA/BRCA1 required for repair of DNA damage [26].

DNA damage biomarkers such as micronuclei (MN), nucleoplasmic bridges (NPBs) and nuclear buds (NBuds) are cytogenetic anomalies measured using the cytokinesis-block micronucleus (CBMN) assay [27]. MN, NPBs and NBuds have been validated with regard to their prospective association with many diseases [28]. It has been shown using the CBMN assay and other biomarkers that nutritional status modifies the extent of DNA damage and DNA integrity [29]. Nutrient deficiency can induce DNA damage because several vitamins and minerals including magnesium play an important role in DNA replication and DNA repair either as substrate and/or as cofactors of key DNA metabolism enzymes [5, 30, 31].

Magnesium plays a critical role in health and development, and wellbeing, however, its role in prevention of DNA damage has not been studied fully in humans so far. Therefore, we tested the hypothesis that magnesium deficiency either on its own or in conjunction with high homocysteine (Hcy) induces DNA damage in vivo in humans.

## Materials and methods

### Recruitment of study participants

Volunteers were recruited through (i) the Commonwealth Scientific and Industrial Research Organisation (CSIRO) Clinical Research Unit database in Adelaide, (ii) a local Channel 7 TV news report of this study and (iii) advertisements posted in hospitals and universities within Adelaide metro area. A total of 172 healthy participants (35–65 years old) were recruited who fulfilled the inclusion criteria: non-smokers, not currently diagnosed with mild cognitive impairment (MCI) or AD, mini-mental state examination (MMSE) score ≥ 20, not on medication for life threatening diseases (e.g. chemotherapy), not taking daily minerals, fish oil or vitamin supplements above the Australian Recommended Dietary Allowance (RDA) level, able to understand the study protocol and not on cholesterol lowering medication. Human Ethics Committee of CSIRO approved the study. Overnight fasted blood samples were collected at the CSIRO clinic by venipuncture. Blood samples were collected between 8.00–10.00am over a period of six months.

### Cytokinesis-Block Micronucleus (CBMN) assay

The assay was performed as described previously [27] with slight modifications and isolated lymphocyte cultures were set up in duplicate. Cultures were incubated for 1 h in a humidified incubator at 37 °C containing 5% CO_2_. Following this incubation, 45µL phytohaemagglutinin (PHA, 22.5 mg/mL; Jomar Diagnostics, Australia) was added to each culture and these cultures were incubated for further 44 h prior to the addition of cytochalasin-B (Cyto-B; Sigma, Sydney, Australia) to a final concentration of 6 µg/mL Following the addition of Cyto-B, the cultures were incubated for another 24 h. Cultured lymphocytes were transferred to TV10 tube containing) and centrifugated at 180 ×g at 20 °C for 10 min. The supernatant was discarded and lymphocytes were re-suspended in 300 µL of RPMI-1640 culture medium containing 5.0 µL dimethyl sulfoxide (DMSO; Sigma Australia) to facilitate disaggregation of cells. Cells were then transferred onto the slides using a cytocentrifuge (Shandon, Runcorn, UK). The air-dried slides were fixed, stained using Diff-Quik (LabAids, Narrabeen, Australia) and scored under code for bi-nucleated (BN) cells containing MN, NPB and NBuds as per previously described scoring criteria [27]. At least 1000 BN cells were scored per slide to determine the frequency of MN, NPB and NBuds in bi-nucleate cells.

### Micronutrient analyses

Blood was collected in 2 mL serum tubes and kept at room temperature (24 °C) for half an hour before being processed by SA Pathology for serum folate analysis. In addition, blood was also collected in lithium heparin tubes for measuring plasma homocysteine and vitamin B_12_ levels. Lithium heparin tubes containing blood were transported to SA pathology on ice. Serum and plasma were separated and analysed on the same day they were collected as per standard protocols by SA Pathology. Serum folate, plasma homocysteine, and vitamin B_12_ concentrations were measured by an Architect® analyser (Abbott Laboratories, IL) in the department of Chemical pathology certified diagnostic laboratory of SA Pathology (Adelaide, South Australia). For magnesium analysis, plasma was isolated from blood collected in lithium heparin tube and stored at -80 °C before analysis. Concentration of total plasma magnesium (Mg) was measured by ICP-MS by Waite Analytical Services, Adelaide, South Australia. The coefficient of variation of duplicate measurements did not exceed 5%.

### Statistical analysis

Parametric statistical methods were used for biomarkers exhibiting Gaussian distribution. Non-parametric methods were employed to analyse the results for biomarkers that do not follow Gaussian distribution. Correlation analysis was performed by Spearman’s or Pearson’s test depending on whether the biomarker data were Gaussian or non-Gaussian in their distribution. The results were adjusted for age and gender. Descriptive statistics were used to summarize demographic characteristics. We also, performed 2-way ANOVA to measure the interactive effects of two factors on a specific biomarker (e.g., effect of Hcy and Mg on MN, NPBs and NBuds). High or low plasma magnesium and Hcy concentration cut off values were based on the median concentrations of the study population (low plasma concentrations were < 19.5 mg/L and < 9.0 µmol/L, and high plasma concentrations were ≥ 19.5 mg/L and ≥ 9.0 µmol/L for magnesium and Hcy, respectively). The normal range value for plasma magnesium in Australian adults aged 18 to < 120 years is 0.70 − 1.10 mmol/L. This range equates to 17.01–26.73 mg/L. Based on the latter normal range we estimate that 94.3% of subjects in our study were within the normal range and 5.7% were deficient (i.e. < 17.01 mg/L in magnesium. The normal range value for plasma homocysteine in Australia for adults is < 15 µmol/L and 99.4% were within this normal range. Statistical tests were performed using Prism 9.0 (Graphpad Inc., USA) and SPSS (IBM SPSS version 23). Significance for all statistical tests was set at *p* < 0.05 for all analyses.

## Results

### Study participants

Table 1 shows the profile of the study participants. There is no significant difference in the ages of males and female cases (mean age 54.78 ± 1.2 and 53.79 ± 0.71 respectively). BMI is marginally higher in males (27.32 ± 0.77) compared to females (26.49 ± 0.49). Similarly, magnesium and Hcy were found to be marginally higher in male participants (19.48 ± 0.21 mg/L; 8.98 ± 0.44 µmol/L respectively) compared to female participants (19.32 ± 0.12 mg/L and 8.65 ± 0.12 µmol/L respectively). Folate and B_12_ were marginally lower in males. MN frequency is significantly higher in females compared to males (19.43 ± 0.75 vs. 13.67 ± 1.45 respectively; *p* < 0.05). However, NPBs and NBuds were found to be marginally lower in males compared to females.

**Table 1:** Baseline characteristics of the study participants

	Male	Female
Number of participants (*n* = 172)	36	136
Mean Age (years)	54.78 ± 1.2	53.79 ± 0.71
BMI (Kg/m^2^)	27.32 ± 0.77	26.49 ± 0.49
Micronuclei (MN)	13.67 ± 1.45	19.43 ± 0.75
Nucleoplasmic bridges (NPBs)	5.52 ± 0.64	5.94 ± 0.33
Nuclear Buds (NBuds)	8.07 ± 0.81	8.33 ± 0.42
Magnesium (mg/L)	19.48 ± 0.21	19.32 ± 0.12
Homocysteine (µmol/L)	8.98 ± 0.44	8.65 ± 0.21
Folate (nmol/L)	33.78 ± 1.52	34.42 ± 0.85
Vitamin B_12_ (pmol/L)	414.3 ± 29.56	420.4 ± 18.21

All values presented are mean ± SEM

### Relationship between Magnesium, Hcy, folate and vitamin B_12_

**Fig. 1:**
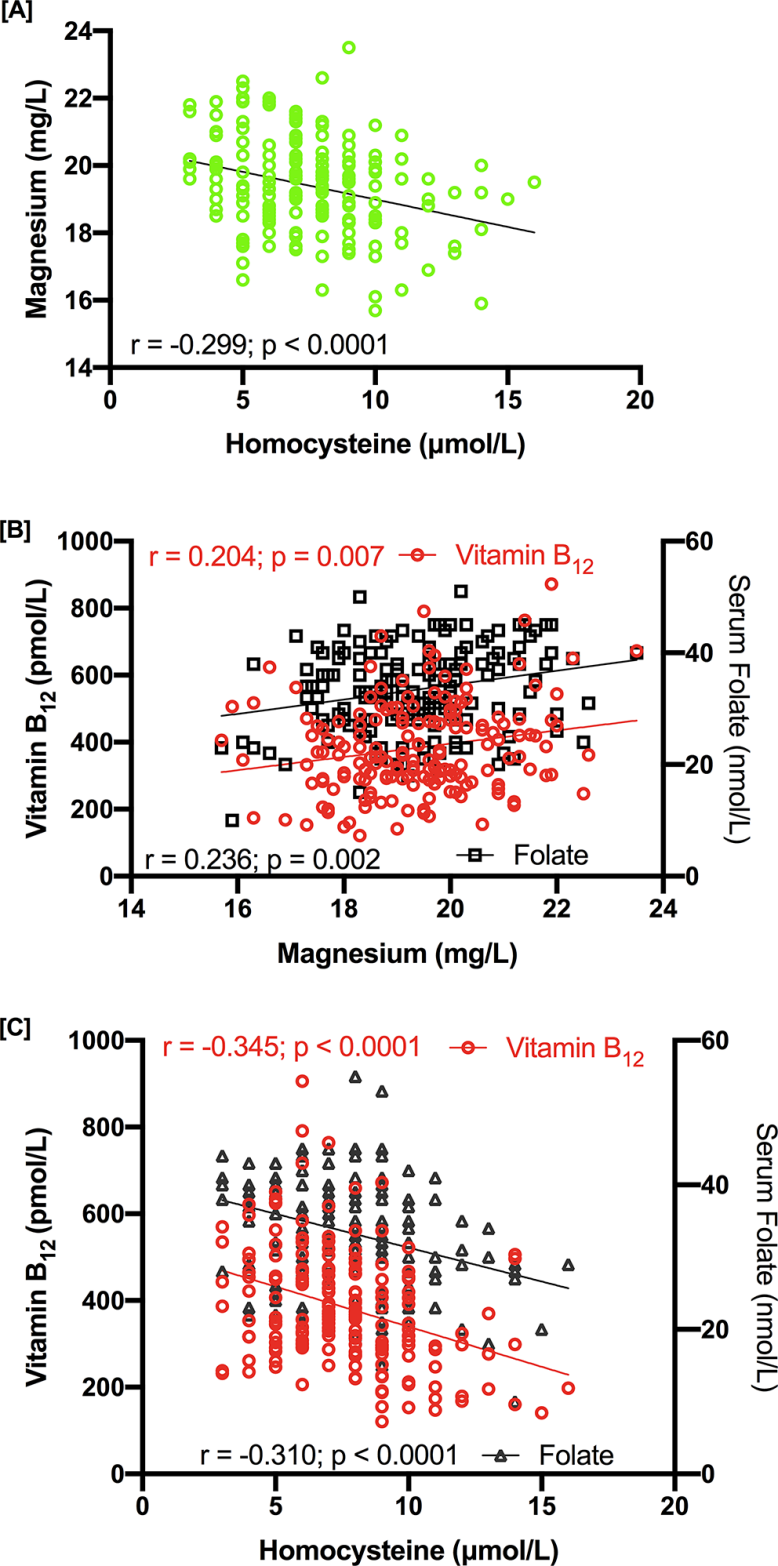
(**A**) The correlation of magnesium with Hcy, (**B**) correlation of vitamin B_12_ (left panel) and folate (right panel) with magnesium, (**C**) correlation of vitamin B_12_ (left panel) and folate (right panel) with Hcy

**Fig. 2:**
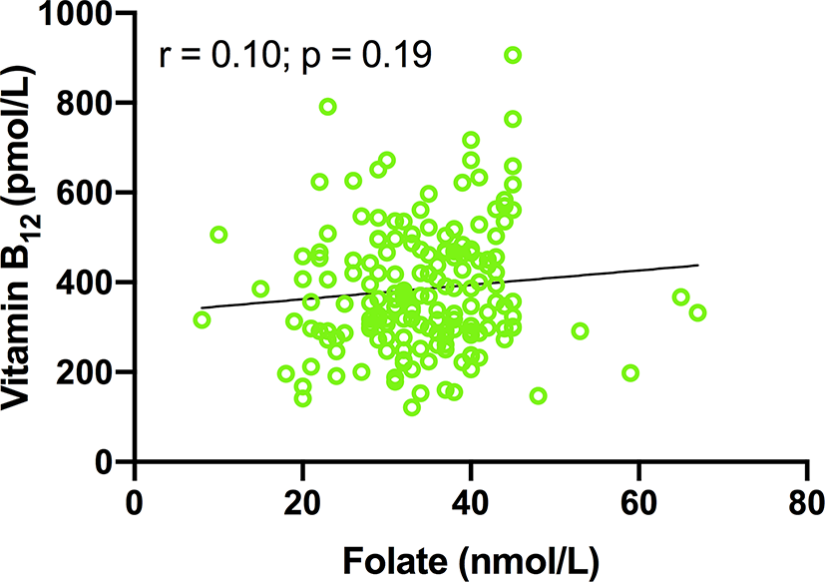
The correlation of vitamin B_12_ with folate

Plasma magnesium is negatively associated with Hcy (*r* = − 0.299; *p* < 0.0001; Fig. 1A). Plasma magnesium concentration shows significant positive correlation with folate (*r* = 0.236; *p* = 0.002; Fig. 1B) and vitamin B_12_ (*r* = 0.204; *p* = 0.007; Fig. 1B). However, Hcy shows a significant inverse correlation with serum folate (*r* = − 0.310; *p* < 0.0001; Fig. 1C), and vitamin B_12_ (*r* = − 0.345; *p* < 0.0001; Fig. 1C). Furthermore, serum folate concentration shows positive correlation with vitamin B_12_ (*r* = 0.10; *p* = 0.19; Fig. 2).

### Correlation of DNA damage biomarkers with Magnesium, Hcy, folate and vitamin B_12_

**Fig. 3:**
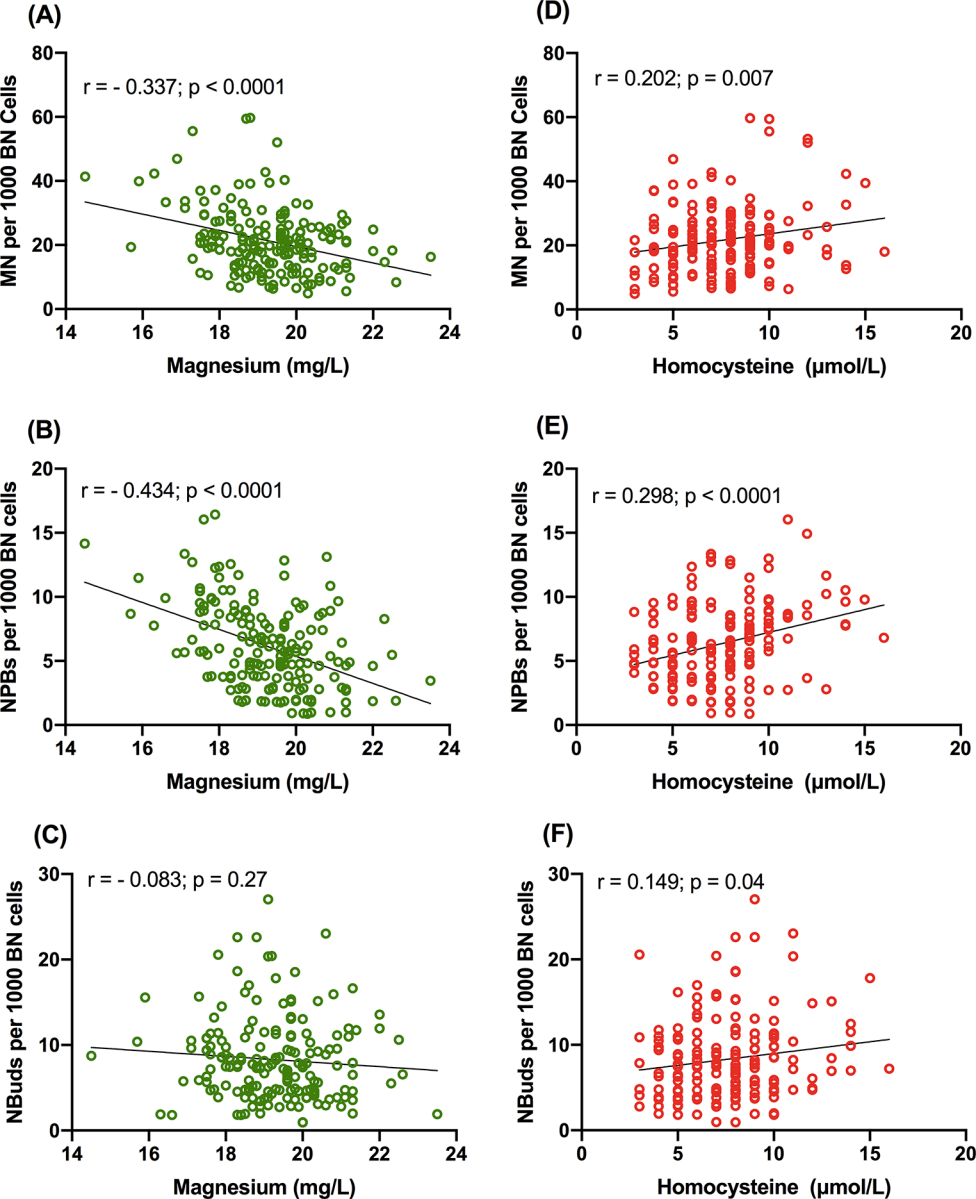
The correlation of CBMN biomarkers with magnesium and Hcy, (**A**) MN with magnesium, (**B**) NPBs with magnesium, (**C**) NBuds with magnesium, (**D**) MN with Hcy, (**E**) NPBs with Hcy and (**F**) NBuds with Hcy

**Fig. 4:**
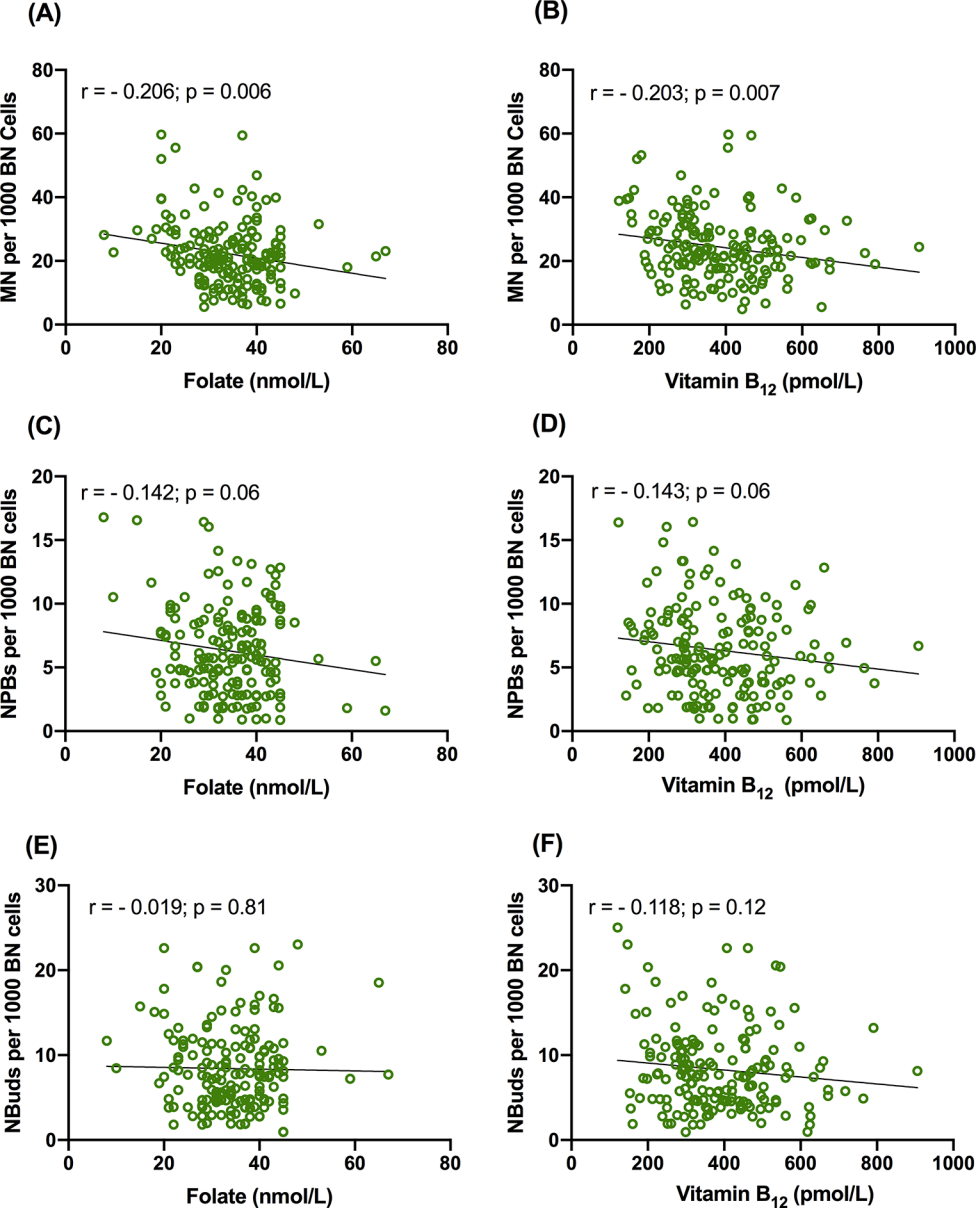
The correlation of CBMN biomarkers with vitamin B_12_ and folate, (**A**) MN with vitamin B_12_, (**B**) NPBs with vitamin B_12_, (**C**) NBuds with vitamin B_12_, (**D**) MN with folate, (**E**) NPBs with folate and (**F**) NBuds with folate

Magnesium is significantly inversely correlated with MN (*r* = − 0.337; *p* < 0.0001; Fig. 3A) and NPB (*r* = − 0.434; *p* < 0.0001; Fig. 3B). NBuds also shows similar trend but it did not reach significance level (*r* = − 0.083; *p* = 0.27; Fig. 3C). Hcy is significantly positively correlated with MN (*r* = 0.202; *p* = 0.007; Fig. 3D), NPB (*r* = 0.298; *p* < 0.0001; Fig. 3E) and NBuds (*r* = 0.149; *p* = 0.04; Fig. 3F). Folate and vitamin B_12_ show significant inverse correlation with MN (*r* = − 0.206; *p* = 0.006 and *r* = − 0.203; *p* = 0.007 respectively; Fig. 4A, B) and not with NPBs (Fig. 4C, D) and NBuds (Fig. 4E, F).

### Effect of Magnesium and Hcy on DNA damage biomarker

**Fig. 5:**
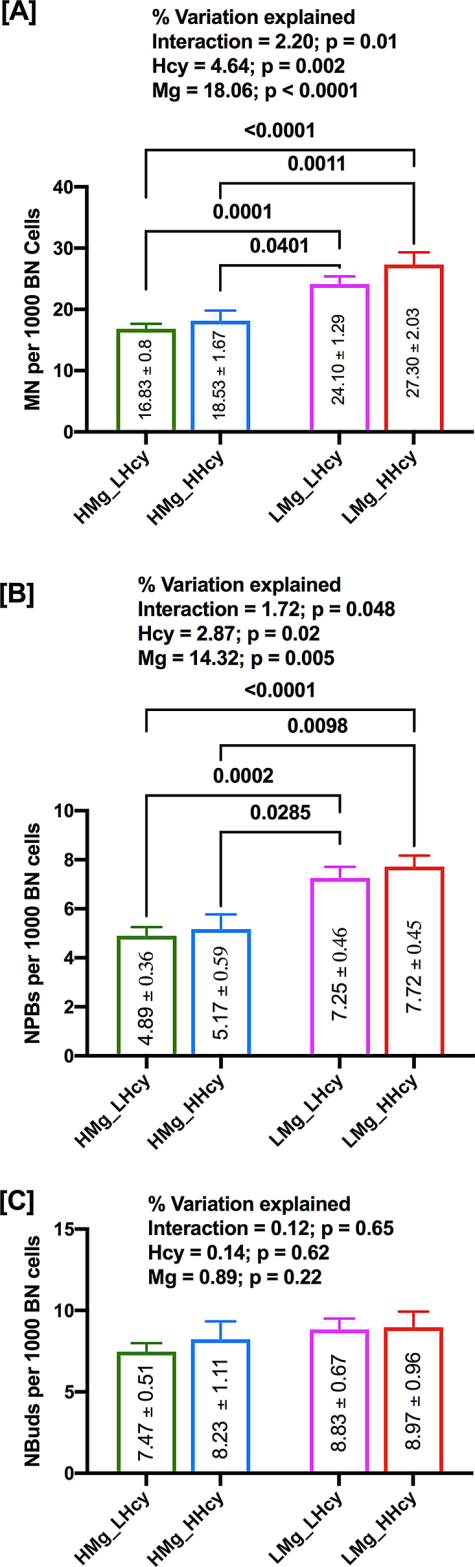
(**A**) Two-way ANOVA analysis of independent and interactive effects of magnesium and Hcy on MN, (**B**) Two-way ANOVA analysis of independent and interactive effects of magnesium and Hcy on NPBs and (**C**) Two-way ANOVA analysis of independent and interactive effects of magnesium and Hcy on NBuds. Mean values of each sub-group for three biomarkers are given in the boxes

Results obtained after 2-way ANOVA indicate that study participants with higher plasma levels of magnesium and low Hcy had lowest frequency of MN (*p* < 0.0001; Fig. 5A). MN frequency was significantly higher in those with low magnesium and high Hcy compared to those with high magnesium and low Hcy (*p* = 0.0001; Fig. 5A). MN frequency was marginally higher in people with high magnesium and high Hcy compared to those with high magnesium and low Hcy. NPB frequency shows similar trend as MN frequency. People with low magnesium and high Hcy had significantly higher NPB frequency compared to those with high magnesium and low Hcy (*p* < 0.0001; Fig. 5B). Participants with low magnesium and low Hcy levels exhibited significantly higher NPB frequency compared to those with high magnesium and low Hcy (*p* = 0.0002; Fig. 5B). Frequency of NBuds was found to be lowest in those participants with high magnesium and low Hcy compared to those with other combinations of magnesium and Hcy, however, NBuds frequency was not found to be significantly different among other groups (Fig. 5C).

### Interaction effects of Magnesium and Hcy on DNA damage biomarkers

The 2-way ANOVA results obtained in the present study indicate significant interactions of magnesium and Hcy with MN frequency (*p* = 0.01; Fig. 5A) and NPBs (*p* = 0.048; Fig. 5B), which explained 2.2% and 1.72% variance respectively, however, there was no significant interactive effect of magnesium and Hcy with NBuds frequency (*p* = 0.65; Fig. 5C).

## Discussion

In the present study, plasma magnesium level was independently and inversely associated with DNA damage biomarkers even after adjusting for covariates such as gender and age. This indicates that higher magnesium levels in blood may protect the genome from endogenous genotoxic events. Killilea and Ames [32] have shown that primary fibroblasts grown in vitro in magnesium deficient medium have accelerated shortening of telomeres and enhanced expression of senescence related biomarkers in addition to loss of their replicative potential. It is possible that chronic magnesium deficiency may result in inducing steady-state oxidative stress as is true for deficiency of other micronutrients such as zinc [1] perhaps by disrupting mitochondrial DNA synthesis and/or antioxidant function by disrupting glutathione synthesis [33]. If it is true, increased oxidative stress caused by low magnesium could result in increased DNA strand breaks and/or oxidation of DNA bases [34]. Results obtained in the present study shows that people with low magnesium have higher DNA damage (i.e. increased number of MN and NPBs). Higher MN frequency may be due to increased unrepaired or mis-repaired DNA breaks that lead to acentric chromosome formation. Mis-repair of DNA breaks also causes the formation of dicentric chromosomes from which NPBs originate when the centromeres are pulled to opposite poles of the cells during anaphase and/or telophase. Magnesium is essential for a vast array of metabolic pathways and its levels like other micronutrients and minerals are constantly in flux. Hence, many homeostatic pathways must accommodate these subtle alterations in magnesium availability to preserve cellular functions like ATP production. Ultimately magnesium deficiency leads to more DNA breaks and loss of acentric fragments, accelerated telomere attrition and genomic instability [15, 35–37]. Although study participants were healthy at the time of sampling, the increased DNA damage in people with low magnesium levels can cause accelerated tissue aging and make them more susceptible to aging related diseases such as Alzheimer’s disease and cancers. Therefore, it is increasingly evident that magnesium plays an important role in protecting against genome damage and telomere attrition as shown in previous reports [15, 38, 39].

Homocysteine (Hcy) is a thiol containing non-proteinogenic amino acid formed during the metabolic conversion of methionine to cysteine in the cell [40]. Deficiency in vitamin cofactors such as folate, vitamin B_6_ and B_12_ or enzymes involved in the folate-methionine pathway specifically in their role as cofactors or substrates of enzymes such as methylenetetrahydrofolate reductase, cystathionine-β-synthase, cystathionase or increased intake of foods containing higher methionine content leads to significant higher levels of Hcy [41–43]. Elevated levels of Hcy (hyperhomocysteinemia) in the body are associated with increased risk for diseases such as cardiovascular diseases, Alzheimer’s disease [44, 45]. In addition, higher levels of Hcy in expecting mothers increases pregnancy complications [46, 47] and has detrimental effects on the developing brain [16, 48]. In the present study we found that MN and NPB frequency is significantly elevated in people with high levels of Hcy. Our findings are in line with previous reports suggesting DNA damaging potential of high Hcy levels [49–53]. It has also been shown that Hcy induces inter-strand cross-links via oxidative stress that can lead to apoptotic cell death [54]. Two recent reports suggest that Hcy in high concentration acts as a pro-oxidant due to its interaction with heme proteins of the cells [55, 56]. Hcy can exert its toxicity through many pathways such as epigenetic dysregulation leading to global hypomethylation, toxic protein modification mainly via irreversible N-homocysteinylation and oxidative stress [55, 57, 58]. It is quite clear that high levels of Hcy induce oxidative stress that can lead to significant increase in DNA damage. Results obtained in the present study clearly points to the possible genotoxic consequences of elevated Hcy.

Post-translational modification of proteins [poly(ADP-ribosyl)ation] plays a critical role in regulating DNA damage repair [59, 60], chromatin structure [61–63] and transcription [63, 64]. Site-specific post-transcriptional modification (PTM) helps in regulating pathways involved in cellular signaling and their regulation is crucial for maintenance of genome integrity [65, 66]. However, its uncontrolled accumulation can lead to cell death. Furthermore, magnesium is required for normal activity of ADP-ribosyl acceptor hydrolase-3 (ARH-3). Recently, it has been shown that ARH3 removes ADP-ribosylation from serine residue [67, 68]. In addition to this, it also cleaves the glycosidic bonds between ADPR unit, thereby hydrolyzing poly(ADP-ribose) chain, suggesting that ARH3 removes PARylation and/or MARylation specifically on serine residue [67–69]. Therefore, this accumulated evidence suggests that dePARylation and deMARylation like PARylation and/or MARylation play an important role in DNA damage repair [70, 71]. Hence it can be assumed that if magnesium concentration is inadequate or deficient, the efficacy of cellular signaling pathways is adversely affected leading to ineffective DNA repair thus resulting in increased DNA damage.

We have previously reported that Hcy is strongly associated with magnesium [15]. A recent report found a significant reduction in Hcy levels on treatment with magnesium sulfate and phentolamine in pregnant women [72]. Hence, we assume that magnesium treatment has been able to lower Hcy levels. Based on these findings, we anticipated lower DNA damage rates in people with high magnesium and low Hcy compared to those with low magnesium and high Hcy concentration in our cohort. Our results indeed indicate that MN and NPB frequency was lowest in those participants who had high magnesium and low Hcy compared to those with other combinations. This is the first study to report that high magnesium levels along with low Hcy are protective in maintaining genome integrity in humans.

**Fig. 6:**
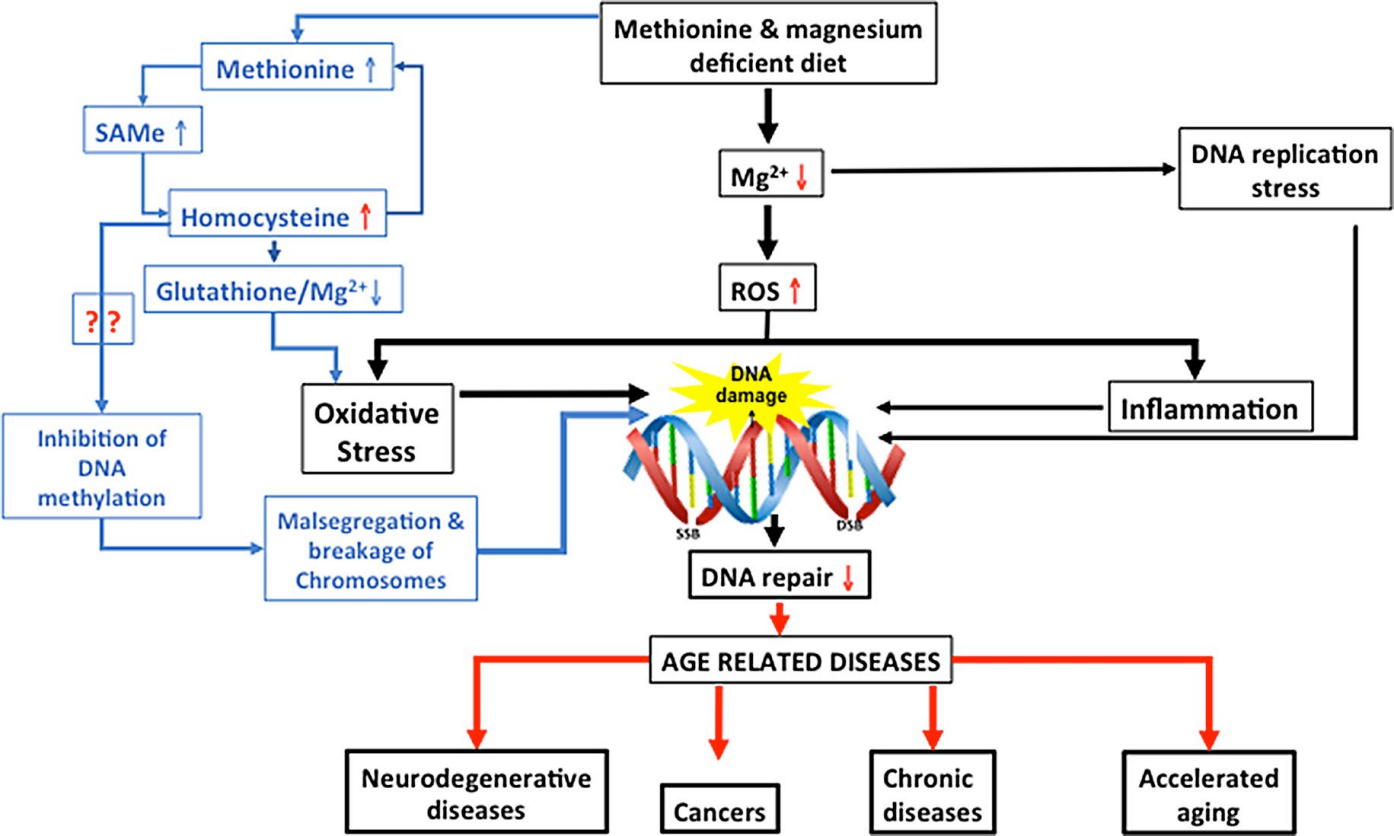
Proposed mechanism of action by which magnesium deficiency and high Hcy cause DNA damage leading to accelerated ageing

Figure 6 summarizes the mechanisms by which magnesium deficiency and high Hcy cause DNA damage and accelerated aging.

## Conclusions

Finally, it can be concluded that low levels of magnesium can have adverse cellular impact by increasing DNA damage rate. Furthermore, low magnesium interacts with high Hcy to increase MN and NPBs which can increase the risk of age-related diseases such as neurodegenerative diseases, chronic diseases, cancers and accelerated aging. In conclusion, the results obtained from our study indicate that optimal intake of micronutrients such as magnesium and B vitamins that can lower Hcy concentration is essential for maintaining genome integrity for healthy ageing. Also, more research is required to determine the optimal dietary intake of magnesium to achieve consistently adequate cellular concentration of magnesium for maintenance of genomic integrity. Furthermore, magnesium dietary requirement may need to consider the homocysteine status of the subjects. Our results support the hypothesis that prevention of magnesium deficiency averts DNA damage measured using the lymphocyte CBMN assay. Whether supplementation with different forms of magnesium (e.g. magnesium citrate or magnesium sulphate) can reduce MN and NPBs requires further study using placebo-controlled trials. The results we present regarding the DNA protection effects of magnesium could benefit clinicians and nutritionists in patient counselling regarding food supplementation, if properly implemented, could impact the onset or progression of cancer and other non-communicable disease.
